# High Mycobacterium tuberculosis Bacillary Loads Detected by Tuberculosis Molecular Bacterial Load Assay in Patient Stool: a Potential Alternative for Nonsputum Diagnosis and Treatment Response Monitoring of Tuberculosis

**DOI:** 10.1128/spectrum.02100-21

**Published:** 2022-01-12

**Authors:** Emmanuel Musisi, Abdul Sessolo, Sylvia Kaswabuli, Josephine Zawedde, Patrick Byanyima, Shariifah Kasinga, Ingvar Sanyu, Esther Uwimaana, Stanley Walimbwa, Joseph Olore, Willy Ssengooba, Christine Sekaggya, Moses L. Joloba, William Worodria, Laurence Huang, Stephen H. Gillespie, Derek J. Sloan, Wilber Sabiiti

**Affiliations:** a Division of Infection and Global Health, School of Medicine, University of St Andrews, Scotland, United Kingdom; b Infectious Diseases Research Collaboration, Kampala, Uganda; c Department of Biochemistry and Sports Sciences, Makerere University, Kampala, Uganda; d Division of Pulmonary and Critical Care Medicine, University of California San Francisco, San Francisco, California, USA; e HIV/AIDS Division, University of California San Francisco, San Francisco, California, USA; f Makerere University Lung Institute, Makerere University, Kampala, Uganda; g Naguru Referral Hospital, Kampala, Uganda; h Department of Medical Microbiology, Makerere University, Kampala, Uganda; i Infectious Diseases Institute, Makerere University, Kampala, Uganda; j Department of Immunology and Molecular Biology, Makerere University, Kampala, Uganda; University of Mississippi Medical Center

**Keywords:** molecular bacterial load assay, molecular diagnostics, *Mycobacterium tuberculosis*

## Abstract

Not all patients produce sputum, yet most available TB tests use sputum. We investigated the utility of a novel RNA-based quantitative test, the tuberculosis molecular bacterial load assay (TB-MBLA), for the detection and quantification of Mycobacterium tuberculosis in stool. Stools from 100 adult individuals were treated with OMNIgene-sputum reagent and tested using Xpert MTB/RIF ultra (Xpert ultra), auramine O smear microscopy (smear), mycobacterial growth indicator tube (MGIT), and Lowenstein-Jensen (LJ) cultures. The remaining portions were frozen at −20°C and later tested by TB-MBLA. MGIT sputum culture was used as a TB confirmatory test and reference for stool tests. Sixty-one of 100 participants were already confirmed TB positive by MGIT sputum culture, 20 (33%) of whom were HIV coinfected. TB-MBLA detected M. tuberculosis in 57/100 stool samples, including 49 already confirmed for TB. The mean bacterial load measured by stool TB-MBLA was 5.67 ± 1.7 log_10_ estimated CFU (eCFU) per mL in HIV-coinfected participants, which was higher than the 4.83 ± 1.59 log_10_ eCFU per mL among the HIV-negative participants (*P* = 0.04). The sensitivities (95% confidence intervals [CI]) of stool assays were 80% (68 to 89) and 90% (79 to 98) for TB-MBLA and Xpert ultra, which were both higher than the 44% (32 to 58), 64% (51 to 76), and 62% (45 to 77) for smear, MGIT, and Lowenstein-Jensen (LJ) stool cultures, respectively. The specificity (95% CI) of stool assays was highest for smear, at 97% (87 to 100), followed by Xpert ultra at 91% (76 to 98), TB-MBLA at 79% (63 to 90), LJ at 80% (64 to 91), and MGIT at 62% (45 to 77). Twenty-six percent of MGIT and 21% of LJ stool cultures were indeterminate due to contamination. Detection and quantification of viable M. tuberculosis bacilli in stool raises its utility as an alternative to sputum as a sample type for TB diagnosis.

**IMPORTANCE** This paper highlights the value of stool as a sample type for diagnosis of tuberculosis. While other studies have used DNA-based assays like the Xpert MTB/RIF and culture to detect Mycobacterium tuberculosis in stool, this is the first study that has applied TB-MBLA, an RNA-based assay, to quantify TB bacteria in stool. The high microbial density and diversity in stool compromises the specificity and sensitivity of culture-based tests due to overgrowth of non-M. tuberculosis flora. Consequently, TB-MBLA becomes the most sensitive and specific test for the detection and quantification of viable TB bacteria in stool. Most crucially, this study raises the possibility of a nonsputum alternative sample type for diagnosis of TB among people who have difficulty in producing sputum.

## INTRODUCTION

Tuberculosis (TB) is a persistent global health challenge (1). In 2020, an estimated 9.9 million people fell ill due to TB. In the same year, an estimated 1.3 million deaths, up from 1.2 million in 2019, occurred among HIV-negative people, and an additional 214,000 deaths occurred among HIV-positive individuals (1). In 2020, WHO reported a sharp reduction in TB case notifications in several high-TB-burden countries, partly due to the COVID-19 pandemic (2). Rapid case detection and treatment initiation is critical to minimizing TB-related morbidity and mortality, especially among vulnerable groups, such as young children and people living with advanced HIV disease. However, diagnosis of TB in young children, the terminally ill, and the immunocompromised, as well as neurologically impaired patients, may be challenging due to inability to provide an adequate sputum sample, leading to low case detection and high mortality rates (3–5). Consequently, bacteriological confirmation of pulmonary TB with microscopy, culture, and Xpert MTB/RIF (Xpert) assay in such groups of individuals may benefit from the use of alternative sample types, such as gastric and nasopharyngeal aspirates (6–9). However, some of these sample collection methods are invasive and have low diagnostic yield (10). Importantly, such sampling procedures are not routinely used in low-resource, high-burden settings. WHO recommends the use of the Xpert MTB/RIF ultra (Xpert ultra) on sputum, nasopharyngeal aspirate gastric aspirates, or stool for the diagnosis of TB in children aged below 10 years (1).

Often, people swallow sputum, which ends up in the gut, and hence, stool has been suggested as an alternative or additional sample type for bacteriological confirmation of pulmonary TB (11–14). The sensitivity and specificity of the Xpert MTB/RIF evaluation of stool varies depending on the population and laboratory processing methods (11, 15–20). The new version, the Xpert MTB/RIF ultra (Xpert ultra), has been shown to have better sensitivity (21); however, Xpert ultra may detect DNA from dead bacilli (22). On the other hand, mycobacterial growth indicator tube (MGIT) stool culture is associated with low yield on stool samples (23).

The tuberculosis molecular bacterial load assay (TB-MBLA) was developed to utilize the abundant cellular 16S rRNA as a marker for the detection and quantification of viable Mycobacterium tuberculosis bacilli in sputum samples. Like viral-load monitoring in HIV-positive individuals, TB-MBLA monitors the TB treatment response by measuring changes in the M. tuberculosis bacillary load over the course of treatment (24–29). Consequently, TB-MBLA potentially offers both diagnostic and treatment response monitoring advantages in real time to inform clinical decision making. A recent multisite study of adult TB patients showed that TB-MBLA is more sensitive than MGIT sputum culture and that its results are reproducible in different settings (30). Additionally, TB-MBLA was proven to differentiate the treatment outcomes of different TB regimens (31). Although TB-MBLA may detect and quantify M. tuberculosis in stool samples, its utility in stool has not been assessed. Having a sensitive quantitative test that can be used on an easily accessible sample may improve TB diagnosis and treatment response monitoring in patients who do not provide suitable sputum samples easily.

In this study, we assessed the ability of TB-MBLA to detect and quantify viable M. tuberculosis bacilli in archived stool samples from presumptive pulmonary TB patients. We show that M. tuberculosis in patient stools was detectable by TB-MBLA with high sensitivity and specificity and that stool could be an alternative sample type for nonsputum diagnosis and treatment response monitoring of TB.

## RESULTS

### Study participants.

Most of the study participants, 54/100, were female adults aged between 33 and 36 years. Of the 100 stool samples tested, 61 samples had already been confirmed positive for pulmonary tuberculosis using MGIT sputum culture. Among the participants who were confirmed positive for pulmonary tuberculosis, 20 (33%) were living with HIV infection, with a median CD4 cell count of 70.5 cells/μL, including 5 (8%) who were on HIV treatment. All participants reported at least one of the following symptoms prior to enrollment: unexplained persistent fever, weight loss, and cough (Table 1).

**TABLE 1:** Demographic and clinical characteristics of the participants who provided stool samples

Characteristic^*a*^	Median value (IQR) or no. (%)			*P* value^*c*^
	Overall (*n* = 100)	Participants with indicated pulmonary TB status^*b*^		
		Positive (*n* = 61 [61%])	Negative (*n* = 39 [39%])	
Age (yr)	34 (25–42)	33 (25–41)	36 (26–45)	0.72
Female	53 (53)	32 (52.5)	21 (53.9)	0.80
HIV positive	36 (35)	20 (33)	16 (41)	0.27
ART use	20 (38)	10 (16.4)	10 (26)	0.31
CD4 cells/μL^*d*^	110 (44–228)	71 (26–171)	170 (66–254)	0.03
BMI	20 (18–22)	19.7 (18–23)	19 (17–21)	
Alcohol use	66 (66)	42 (69)	24 (61)	0.63
Smoking	21 (21)	12 (20)	9 (23)	0.73
Fever	79 (79)	48 (78.7)	31 (79)	0.86
Wt loss of >5%	87 (87)	54 (88.5)	33 (84)	0.78
Cough for >2 wks	100 (100)	61 (100)	39 (100)	0.46
HR	100 (84–111)	100 (84–111)	101 (81–111)	
RR	22 (20–26)	22 (20–26)	24 (20–27)	

a HIV, human immunodeficiency virus; ART, antiretroviral therapy; BMI, body mass index; HR, heart rate; RR, respiratory rate.

b Bacteriologically confirmed positive or negative TB cases.

c Comparison between pulmonary-TB-positive and -negative participants.

d Measured for HIV-infected adults only (*n* = 36).

### Stool M. tuberculosis bacillary load quantification.

**FIG 1:**
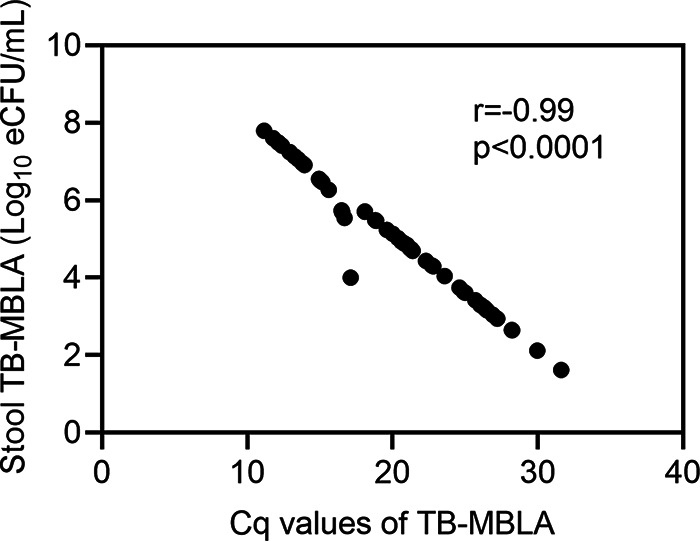
Correlation between the TB-MBLA *C_q_* and bacterial load values in stool samples. The molecular bacterial loads (log_10_ CFU/mL) and the *C_q_* values showed a strong negative correlation (*r* = −0.99).

Measured as the conversion of quantification cycle (*C_q_*) values to CFU per mL, the overall mean bacterial load (mean ± standard deviation [SD]) in stool was 5.1 ± 1.59 log_10_ estimated CFU (eCFU) per mL. Stool from participants living with HIV had a mean bacterial load of 5.67 ± 1.7 log_10_ eCFU per mL, which was higher than the 4.83 ± 1.59 log_10_ eCFU per mL in stool of HIV-negative participants (*P* = 0.04). In line with previously published data for sputum TB-MBLA (25), the *C_q_* values and the molecular bacterial loads measured by the stool TB-MBLA were inversely related (*r* = −0.99) (Fig. 1). To compare the quantification between stool TB-MBLA and stool Xpert ultra, samples that were positive by both assays were considered. The mean cycle threshold (*C_T_*) values of all the stool Xpert ultra probes were calculated and compared with the *C_q_* values of the stool TB-MBLA. A Mann-Whitney test showed that the median *C_q_* value of 20.3 (interquartile range [IQR], 15.4 to 24.8) for the stool TB-MBLA was significantly lower than the median *C_q_* value of 25.1 (IQR, 22 to 28) for stool Xpert ultra (*P* < 0.00001; *n* = 45). Importantly, we note that equal volumes of stool sample were used for TB-MBLA and Xpert ultra. A Spearman’s correlation analysis showed a nonsignificant positive correlation between the stool TB-MBLA and stool Xpert ultra *C_T_* values (*r* = 0.17, *P* = 0.25).

### Sensitivity and specificity of stool assays with reference to MGIT sputum culture.

Using MGIT sputum culture as the reference test, the sensitivity (95% CI) of stool assays was 80% (68 to 89) for TB-MBLA and 90% (79 to 98) for Xpert ultra, and both were higher than the 44% (32 to 58) for smear, 64% (51 to 76) for MGIT, and 62% (45 to 77) for LJ stool cultures. The specificity (95% CI) of stool assays was highest for smear at 97% (87 to 100), followed by 91% (76 to 98) for Xpert ultra, 79% (63 to 90) for TB-MBLA, 80% (64 to 91) for LJ, and 62% (45 to 77) for MGIT (Table 2).

**TABLE 2:** Results for analysis of accuracy of stool assays

Test^*a*^	Mean value (95% CI) for^*b*^:			
	Sensitivity	Specificity	PPV	NPV
TB-MBL^*c*^	80 (68–89)	79 (63–90)	86 (74–93)	72 (56–85)
Xpert ultra	90 (79–98)	91 (76–98)	86 (70–95)	86 (70–95)
FM smear	44 (32–58)	97 (87–100)	96 (82–100)	53 (41–65)
MGIT culture	64 (51–76)	62 (45–77)	52 (37–67)	52 (37–67)
LJ culture	44 (32–58)	80 (64–91)	48 (35–61)	48 (35–61)

a TB-MBLA, tuberculosis molecular bacterial load assay; Xpert ultra, Xpert MTB/RIF ultra; FM, fluorescence microscopy; MGIT, mycobacterial growth indicator tube; LJ, Löwenstein-Jensen.

b MGIT sputum culture was used as the reference. PPV, positive predictive value; NPV, negative predictive value.

c Done using stool frozen at −20°C for 18 months.

### Concordance between stool assays and MGIT sputum culture.

**FIG 2:**
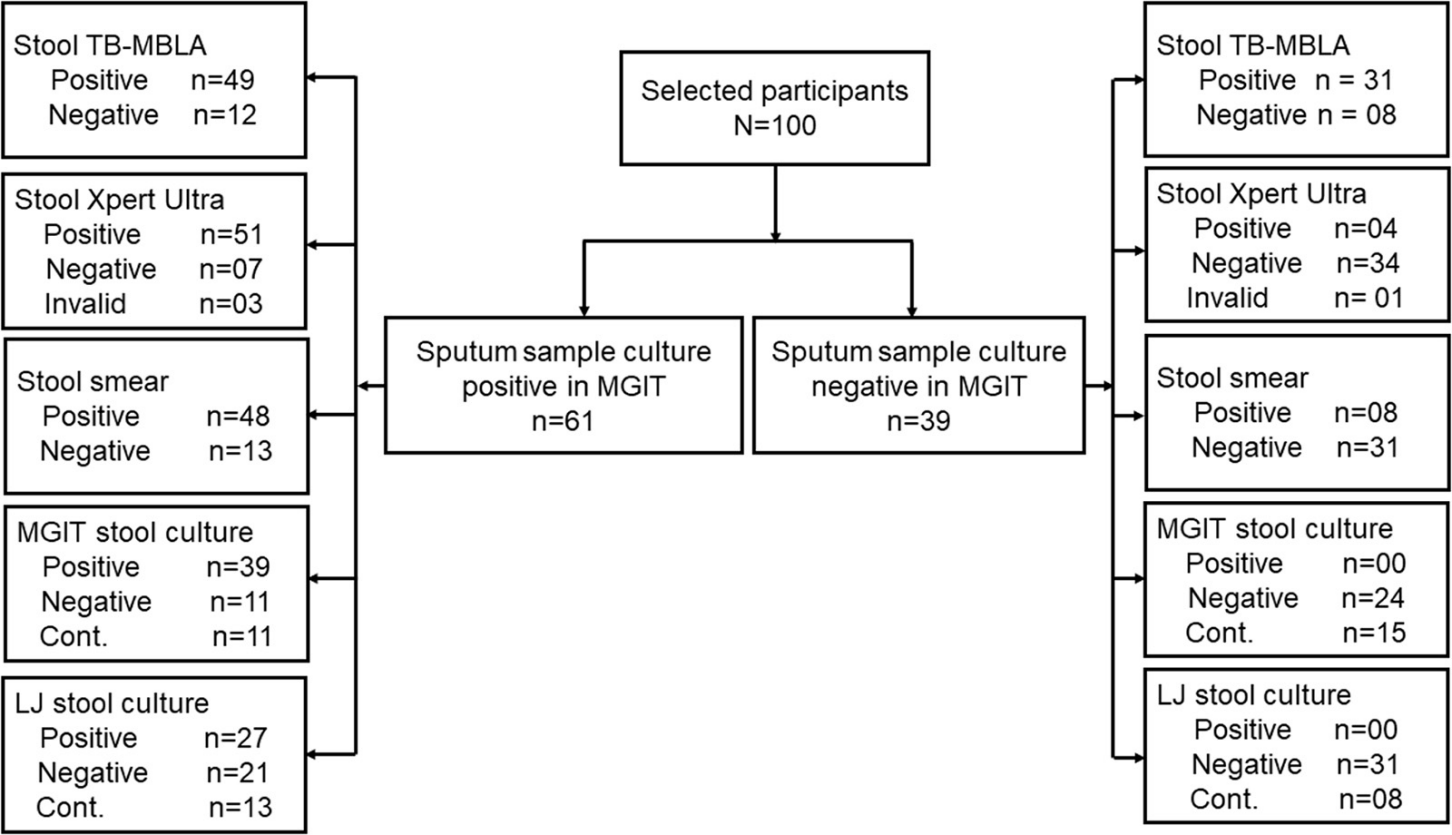
Flow chart showing the numbers of patients, samples, and test results for stool samples. TB-MBLA, tuberculosis molecular bacterial load assay; Xpert, Xpert MTB/RIF ultra; smear, smear fluorescence microscopy; MGIT, mycobacterial growth indicator tube; LJ, Löwenstein-Jensen culture.

Sixty-one of 100 of the tested participants were confirmed positive for pulmonary tuberculosis (PTB) by MGIT sputum culture. TB-MBLA detected and quantified M. tuberculosis in 57/100 participants, 49 of whom were among the 61 participants that had already been confirmed PTB positive by MGIT sputum culture. Stool Xpert ultra identified M. tuberculosis in 55/100 of the tested samples, of which 51/100 had been confirmed by MGIT sputum culture. Stool LJ culture detected the lowest number, 27 of the 61 MGIT sputum-positive cultures (Fig. 2). The overall percentages of concordance of stool positivity and negativity to MGIT sputum culture results were higher with molecular assays than with nonmolecular assays. Using kappa analysis, the two molecular assays strongly agreed with MGIT sputum culture, at 81% (κ = 0.6) for TB-MBLA and 87% (κ = 0.72) for Xpert ultra. Among the nonmolecular assays, stool smear had the highest concordance with the MGIT sputum culture, at 63% (κ = 0.34), followed by MGIT stool at 46% (κ = 0.2) and LJ stool at 42% (κ = 0.2), respectively (Table 3).

**TABLE 3:** Results for analysis of the concordance of stool assays and MGIT sputum culture results

Test^*a*^	% agreement			κ statistic	Strength
	Positive	Negative	Overall		
TB-MBLA	82	89	84	0.67	Substantial
Xpert ultra	85	90	87	0.72	Substantial
MGIT culture	62	60	61	0.2	Poor
LJ culture	43	80	56	0.19	Poor
FM smear^*b*^	42	97	61	0.31	Fair

a TB-MBLA, tuberculosis molecular bacterial load assay; Xpert ultra, Xpert MTB/RIF ultra; MGIT, mycobacterial growth indicator tube; LJ, Löwenstein-Jensen; FM, fluorescence microscopy.

b Done using stool frozen at −20°C for 18 months.

### Concordance within TB-positive stool assays.

**FIG 3:**
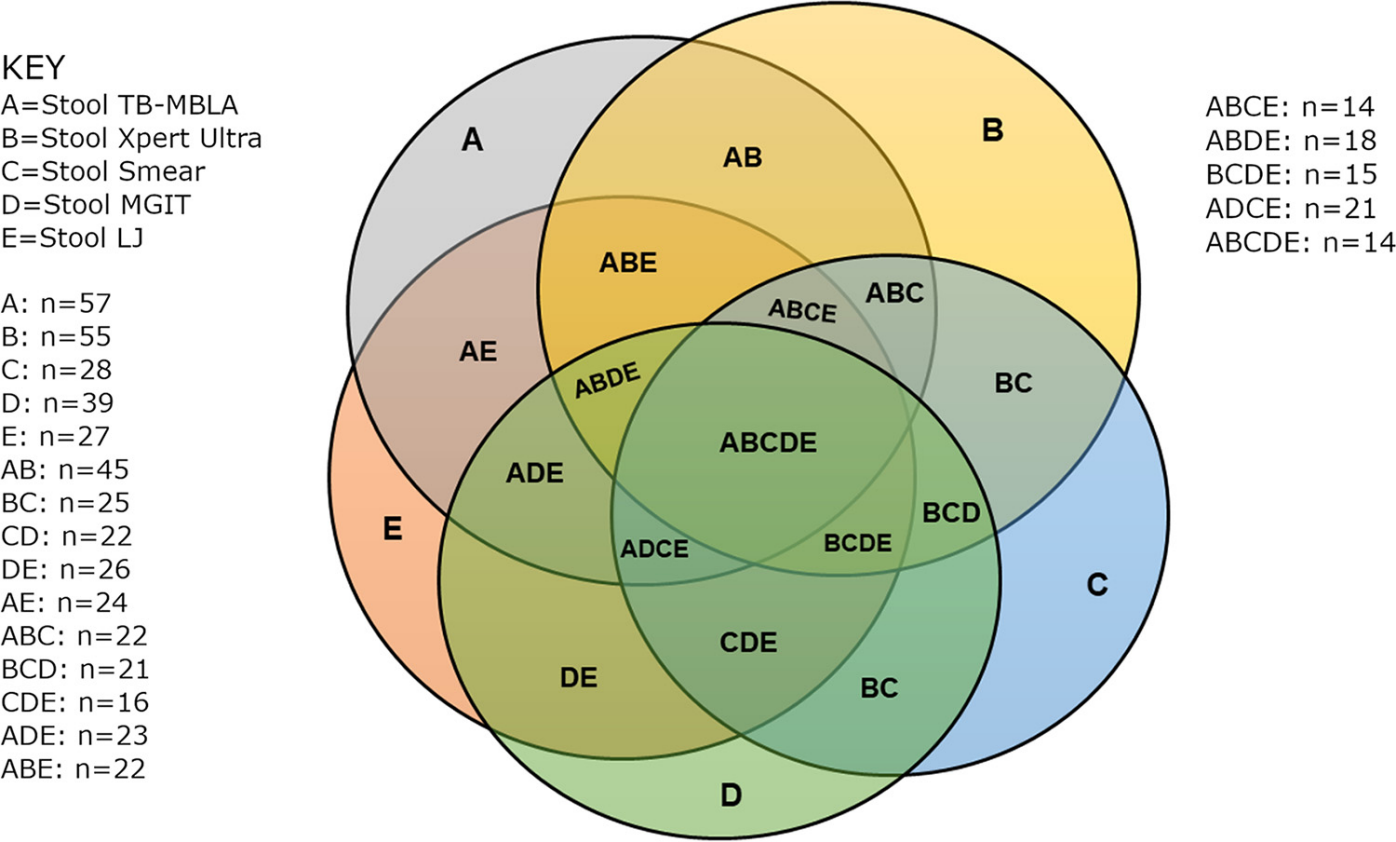
Venn diagram for positive results for stool assays. A, TB-MBLA (*n* = 57); B, Xpert ultra (*n* = 55); C, smear (*n* = 28); D, MGIT culture (*n* = 39); E, LJ culture (*n* = 27); AB, TB-MBLA and Xpert ultra (*n* = 45); BC, Xpert ultra and smear (*n* = 25); CD, smear and MGIT culture (*n* = 22); DE, MGIT culture and LJ culture (*n* = 26); AE, TB-MBLA and LJ culture (*n* = 24); ABC, TB-MBLA, Xpert ultra, and smear (*n* = 22); BCD, Xpert ultra, smear, and MGIT culture (*n* = 21); CDE, smear, MGIT culture, and LJ culture (*n* = 16); ADE, MGIT culture, LJ culture, and TB-MBLA (*n* = 23); ABE, TB-MBLA, Xpert ultra, and LJ culture (*n* = 22); ABCE, TB-MBLA, Xpert ultra, smear, and LJ culture (*n* = 14); ABCD, TB-MBLA, Xpert ultra, smear, and MGIT culture (*n* = 18); BCDE, Xpert ultra, smear, MGIT culture, and LJ culture (*n* = 15); ABDE, TB-MBLA, Xpert ultra, MGIT culture, and LJ culture (*n* = 21); ABCDE, TB-MBLA, Xpert ultra, smear, MGIT culture, and LJ culture (*n* = 14). It is apparent that molecular assays detect more TB-positive cases than do smear and culture tests and that combining molecular assays with stool culture or stool smear microscopy does not increase the number of identified TB cases, meaning that one molecular test is sufficient for a clinician to make a clinical decision.

Using a Venn diagram, we illustrated the levels of concordance within stool assays. Considering the samples detected as TB positive by each test, we found that only 14/100 were consistently positive by all five stool tests. Concordance was highest between the two molecular tests, stool TB-MBLA and Xpert ultra, with agreement for 45/100 samples tested. Molecular tests and combined stool MGIT and LJ cultures showed the 2nd highest concordance, with agreement for 21/100 samples tested. Concordance was only 14/100 in a combination of stool TB-MBLA, Xpert ultra, smear, and LJ culture (Fig. 3).

### Indeterminate stool culture.

We noted that 26% and 21% of MGIT and LJ stool cultures, respectively, were contaminated, and their results were indeterminate (neither positive nor negative status) (Table 4). Twelve (46%) of the indeterminate MGIT stool cultures were positive by both stool TB-MBLA and stool Xpert ultra. Considering the LJ stool cultures, 14 (67%) of the indeterminate samples were positive by stool TB-MBLA, while 12 (57%) were positive by stool Xpert ultra. Overall, indeterminate results that were resolved by stool TB-MBLA and stool Xpert ultra were concordant with MGIT sputum culture at 81% (κ = 0.54) and 85% (κ = 0.73), respectively, suggesting that they were true positives (Table 4). In contrast, we observed weak positivity and negativity concordance of the results that were resolved by stool smear and MGIT sputum culture, at 43% (κ = 0.2) and 62% (κ = 0.2), respectively, indicating the possibility of false positivity by stool smear microscopy.

**TABLE 4:** Results for analysis of the indeterminate results in stool samples that were resolved by TB-MBLA and Xpert ultra

Test^*a*^	No. (%) with indeterminate culture result^*b*^	No. (%) with indicated result in^*c*^:					
		TB-MBLA		Xpert ultra		Smear	
		Positive	Negative	Positive	Negative	Positive	Negative
MGIT culture	26 (26)	12 (46)	14 (54)	12 (46)	14 (54)	2 (8)	24 (92)
LJ culture	21 (21)	14 (67)	7 (33)	12 (57)	9 (43)	5 (24)	16 (76)

a MGIT, mycobacterial growth indicator tube; LJ, Lowenstein-Jensen.

b Contaminated culture results that were neither positive nor negative.

c TB-MBLA, tuberculosis molecular bacterial load assay; Xpert ultra, Xpert MTB/RIF ultra.

### Stool TB-MBLA-positive but sputum MGIT-negative participants.

Eight patient stool samples were positive by TB-MBLA, but their corresponding MGIT sputum cultures were negative. Further analysis revealed that 6 of these 8 stool samples had at least one positive corresponding sputum test: 4 of them were positive by both sputum smear and sputum Xpert ultra and 2 were positive by only sputum smear but negative by sputum Xpert ultra. The remaining 2 stool samples were negative by all the investigated sputum tests.

### Stool TB-MBLA-negative but sputum MGIT-positive participants.

Twelve patient stool samples tested negative by TB-MBLA, but their corresponding MGIT sputum cultures were positive. Compared with the corresponding stool Xpert ultra-tested samples, 6 samples were negative, 5 were positive and detected as “low” (*n* = 4) and “trace” (*n* = 1), and 1 was invalid. The mean MGIT time to positivity (TTP) of the sputa that corresponded to the stool samples that were negative by both stool TB-MBLA and stool Xpert ultra was 11 days, indicating a moderately high bacillary load in sputa. The mean *C_q_* value of the extraction control (±SD) in stool samples that were positive by stool TB-MBLA was 24.1 ± 1.9, and for the negative stool samples, it was 24.1 ± 2.3, not a significant difference (*P* = 0.48), potentially indicating absence of inhibition. Additionally, the average *C_q_* value for 7/12 stool samples was 0, indicating the absence of any amplification by TB-MBLA, but 5 of the samples showed late amplification with an average *C_q_* value of 32.8, well above the limit of detection of 30 *C_q_*.

### Time to positivity and *C_q_* values.

**FIG 4:**
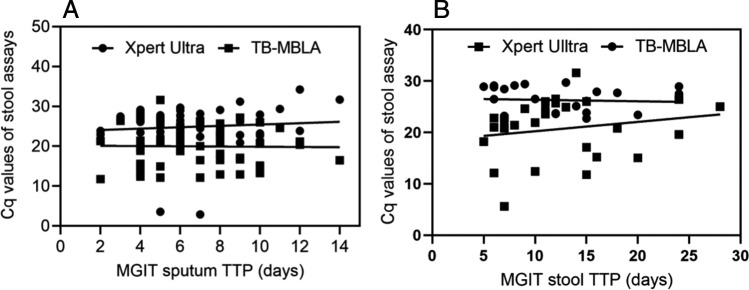
(A) Relationship between the *C_q_* values of stool TB-MBLA or the *C_q_* values of stool Xpert ultra and the MGIT sputum culture time to positivity (TTP) (days). The Spearman regression *R*^2^ values were 0.000 and 0.13 for stool TB-MBLA and stool Xpert ultra, respectively. (B) Relationship between the *C_q_* values of stool TB-MBLA or the *C_q_* values of stool Xpert ultra and the MGIT stool culture TTP (days). The Spearman regression *R*^2^ values were 0.02 and 0.04 for stool TB-MBLA and stool Xpert ultra, respectively. Overall, we did not find a significant correlation between *C_q_* values and MGIT TTP.

Overall, the mean MGIT TTP ± SD of 12.3 ± 6.4 days in stool samples was higher the 7.1 ± 3.1 days in sputum samples. Prior work on sputum TB-MBLA (25) showed a strong, direct correlation between *C_q_* values and MGIT TTP. We thus investigated whether this was replicated in stool TB-MBLA and found that the mean *C_q_* values for both stool TB-MBLA and stool Xpert ultra did not significantly correlate with the MGIT sputum TTP. Similarly, there was no significant relationship between *C_q_* values and MGIT stool culture TTP (Fig. 4A and B).

## DISCUSSION

Stool is an easy-to-obtain sample and could enhance TB diagnosis in individuals who cannot provide adequate sputum samples, and yet, MGIT culture, which is the current confirmatory test for TB, has low yields for stool. In this study, we assessed the ability of a molecular-based fully quantitative assay, the TB-MBLA, to detect and quantify viable M. tuberculosis bacilli in frozen stool samples.

We show that TB-MBLA for stool is sensitive and specific, signifying its potential utility for clinical decision making. Our results provide evidence for the presence of high bacillary loads in stool samples both in people living with HIV and those without, indicating its potential broad applicability in multiple settings. It is not clear whether the higher bacillary loads observed in stool samples of HIV-positive patients are due to higher dissemination of bacilli from the respiratory tract to the gut. A larger-scale study would establish the robustness of this difference and what mechanisms underlie it.

Although TB-MBLA had significantly lower *C_q_* values (potentially higher bacterial loads detected), Xpert ultra had higher sensitivity and specificity than TB-MBLA. The lower TB-MBLA *C_q_* values could be explained by the fact that TB-MBLA detects the more abundant rRNA compared to the DNA detected by Xpert MTB/RIF. Mycobacterium tuberculosis has been shown to contain 700 ribosomes per cubic micrometer of cytoplasm, implying a larger amount of rRNA available for detection (32). Ideally, this would mean higher sensitivity of TB-MBLA than of Xpert ultra, but the reverse was true. One reason could be the use of multicopy M. tuberculosis gene targets in Xpert ultra, enabling it to detect fewer bacilli in a sample and to have a lower limit of detection (33). Furthermore, unlike Xpert ultra, specimen processing for TB-MBLA, particularly the initial centrifugation step to harvest M. tuberculosis cells from sputum, has been shown to cause a substantial loss of viable M. tuberculosis bacilli, which do not easily sediment, and consequently reduces assay sensitivity (34). Third, loss of M. tuberculosis cell viability due to prolonged storage at −20°C may have compromised the sensitivity of TB-MBLA.

In principle, there should be a correlation between rRNA and DNA detected in the same cell. However, our analysis showed a nonsignificant correlation between stool TB-MBLA and Xpert ultra *Cq* values. This requires further analysis in a large-scale study ensuring all analyses are done on freshly collected samples to eliminate the confounder of loss of viability caused by storage conditions. Second, the average *C_q_* value for Xpert MTB/RIF should be derived from probes that detect M. tuberculosis DNA and not those of the non-TB internal controls of the assay.

Overall, our results concur with findings from other studies where molecular assays were shown to have higher diagnostic accuracy than stool culture assays when MGIT sputum culture was used as the reference test (35, 36). The low diagnostic accuracy of stool culture may partly be attributed to overgrowth of non-M. tuberculosis flora due to the rich microbial population in the gut, while loss of M. tuberculosis viability during transition in the gut is plausible but requires further investigation, since substantial numbers of the contaminated stool cultures were detected as M. tuberculosis positive by TB-MBLA, which is known to detect viable M. tuberculosis bacilli. M. tuberculosis growth inhibition and/or reduction of viable bacillary load due to the killing effect of the decontamination methods could be a factor in reducing the productivity of culture and further highlights the importance of sample processing methods (26).

In this study, stool samples were processed using OMNIgene-sputum (OM-S) reagent. OM-S suppresses contaminants, but it delays MGIT sputum time to culture positivity (TTP) (37, 38). It is plausible that the same effect occurred in our stool samples; hence the absence of correlation between MGIT TTP and the *C_q_* values of the molecular tests. Other studies have shown inverse correlation of MGIT TTP and *C_q_* values (24, 27, 39).

We noted that 12 samples from PTB cases confirmed by MGIT sputum culture were not amplified by stool TB-MBLA. Whether this was due to inhibition could not be ascertained, because the spiked extraction control was efficiently recovered, as demonstrated by quantitative PCR (qPCR) amplification. However, we hypothesize that loss of M. tuberculosis viability during stool storage at −20°C, compounded by some loss of RNA during extraction, may have led to nondetection by TB-MBLA (34).

We also observed that in 5 of the 12 TB-MBLA-negative samples, the Xpert ultra positivity results were low/trace, implying that there were few bacilli in the samples prior to storage at −20°C. Some stool samples showed late amplification, and these reported as negative based on the cutoff value of 30. Whether the *C_q_* cutoff of 30 wrongly placed these samples in the “TB-negative” category could not be ascertained. Future studies need to investigate the *C_q_* cutoff value for stool.

Using molecular-based assays for stool has the potential to increase TB case detection in both adults and children, because they use M. tuberculosis-specific primers and probes that are not affected by non-M. tuberculosis flora present in stool. In this study, we show that 26% of the tested stool samples were contaminated (grew non-TB flora) on MGIT stool culture and 21% on LJ stool culture. All contaminated culture samples had a definitive (positive or negative) result by TB-MBLA. To confirm the validity of these results, the percentages of agreement with the reference standard test were calculated and found to range between 81 and 85%, implying that over 80% of the resolved results were valid and could be used to inform clinical decision making. Unlike Xpert ultra, which may detect nonviable M. tuberculosis bacilli due to persistent DNA after cell death, TB-MBLA has been shown to be sensitive to agents that reduce cell viability (30, 40).

The RNA extraction part of TB-MBLA is manual and takes a substantial amount of hands-on time and, thus, would benefit from automation of the process to increase its ease of implementation in the clinical laboratory setting. It is important to note that even in its current form, TB-MBLA is more rapid than culture. Shortening the TB-MBLA protocol will increase its potential for real-time application in the real-time clinical setting.

Diagnosis in children and immunocompromised individuals is difficult, usually depending on the unreliable clinical symptoms. Using stool samples and TB-MBLA could improve the confidence of clinicians in deciding to initiate or withhold treatment. Placing patients on an appropriate treatment early enough minimizes the risk of TB transmission and associated mortality. Smear microscopy is the fastest and the most accessible test, making it the most common TB diagnostic in resource-constrained settings (41). However, as in sputum (42), we noted that its sensitivity in stool was lower than that of molecular tests and that it might not give reliable yields if performed for a sample that had less than 10,000 bacilli/mL, as also reported elsewhere (43, 44). The detection of substantial bacillary loads in both HIV-negative and -positive stool samples is a testament to the sensitivity of stool as a sample type and makes it worth considering among sample types for primary diagnosis of TB. Further studies should investigate the mechanism underlying the higher bacillary loads in HIV-positive than in HIV-negative patients.

Using stool samples might provide an easier way to enhance diagnosis of gastrointestinal TB. Gastrointestinal TB is a life-threatening form of TB that control programs have not accorded due attention, perhaps because it is less transmissible and is challenging to diagnose (45). We noted that TB-MBLA detected and quantified M. tuberculosis in 8 stool samples among the MGIT sputum-negative participants, 2 of which did not have any corresponding positive sputum test. Whether these cases represented gastrointestinal TB or not could not be ascertained in this study because we lacked data about the relevant descriptors, such as colitis symptoms and Bristol stool chart scales. Future evaluations to unravel the use of stool specimens to diagnose gastrointestinal TB are urgently necessary.

### Limitations.

The main limitations of this study are the small sample size and the use of stored samples for TB-MBLA. Storing samples at −20°C for 18 months could have degraded some rRNA, hence lowering the sensitivity of TB-MBLA. Nevertheless, we demonstrate the ability of TB-MBLA to detect and quantify M. tuberculosis in stool with a high level of accuracy, indicating its potential role in enabling clinical decision making. Besides, TB-MBLA is rapid and will enable rapid detection and quantification of M. tuberculosis in clinical specimens. Molecular testing of stool may facilitate TB diagnosis in patients unable to produce sputum and in screening of high-risk groups.

Future studies will explore the diagnostic accuracy and treatment response monitoring utility of TB-MBLA in a larger sample size of fresh stool samples from patients who are unable to provide sputum, including children. These investigations will benefit from modifying the current sample-processing methods to make it shorter and also to minimize the loss of bacilli caused by centrifugation steps. Future studies will also explore the role of TB-MBLA for revealing the mycobactericidal effect of TB regimens in patient populations where sputum production is often problematic, including children.

## MATERIALS AND METHODS

### Ethics.

The parent and current studies were approved by the Makerere University School of Medicine Research and Ethics Committee (REC reference no. 2006-017) and the Makerere University School of Biomedical Sciences Research and Ethics Committee (REC reference no. SBS 529), respectively. All patients consented to the use of their stored stool samples and data for future TB investigations. Good clinical practice guidelines were observed (46).

### Study site and design.

We conducted a cross-sectional, laboratory-based study utilizing banked stool samples collected between 2018 and 2019. The study was nested in a cohort of persons with pneumonia (47–49) at Naguru Referral Hospital, Kampala, Uganda.

### Specimen collection and processing.

At enrollment, participants provided spot stool (∼12.0 g) in a prelabeled sterile wide-mouthed container (Sarstedt, Australia). Six grams of each stool sample was homogenized and incubated at ambient temperature in OMNIgene-sputum reagent (OM-S; DNA Genotek, Inc., Ottawa, Canada) for 10 min. OM-S reagent decontaminates TB samples while preserving M. tuberculosis viability (50). The resulting suspension was pelleted twice in OM-S at 3,000 × *g*, for 20 and 10 min, respectively. The resulting pellet was resuspended in 6 mL of phosphate-buffered saline before being aliquoted into four different portions of 1 mL each to be tested by auramine O smear microscopy (smear), Xpert MTB/RIF ultra (Xpert ultra), MGIT, and LJ cultures. The remaining portions were banked at −20°C and tested by TB-MBLA in a batch on all specimens at once. OM-S reagent is compatible with TB molecular assays, smear, and solid and liquid culture tests (51, 52). In addition to stool samples, sputum samples were collected and tested on the same day using MGIT sputum culture and Xpert ultra. We note that sputa were not processed in OM-S but, rather, decontaminated using 2% NaOH/*N*-acetyl l-cysteine.

### Selection of banked stool samples.

By the time of this study, there were 600 stored stool samples, 597 of which had a valid corresponding MGIT sputum culture result as the reference comparator and all of which had valid Xpert results on sputum as the standard-of-care test. A systematic random sampling selection method (52) was conducted to obtain 100 archived stool samples. A sampling interval was calculated as the ratio of total banked stool samples to the target sample size. Samples without matching clinical data, sputum Xpert ultra, and MGIT sputum culture results were excluded. We note that selection of the samples was not based on any stool-related results.

### TB-MBLA.

Total RNA was extracted by adopting a method described elsewhere (24, 25, 27). Homogenized stool samples (1 mL) were thawed at ambient temperature, spiked with 100 μl of the extraction control, and then centrifuged for 15 min at 3,000 × *g*. RNA was isolated using the Fast Prep RNA pro blue kit (MP Biomedicals, Santa Ana, CA, USA). Duplex reverse transcriptase qPCR targeting both Mycobacterium tuberculosis and the extraction control was performed on a Rotor-Gene 5plex platform (Qiagen, Manchester, UK). Primers and TaqMan dually labeled probes were manufactured by MWG Eurofins, Germany. qPCR cycling conditions were as reported by Honeyborne et al. (24). Quantification cycle (*C_q_*) values were converted to bacterial loads using a standard curve customized for the site’s qPCR platform and recorded as estimated CFU per mL (eCFU/mL) (25). The standard curve was constructed using M. tuberculosis rRNA that was extracted from M. tuberculosis culture of a known concentration (CFU/mL). Stool samples without *C_q_* values and those with *C_q_* values above 30 were reported as TB negative.

### Stool Xpert ultra.

One milliliter of the homogenized stool was mixed with 2 mL of the sample reagent buffer and tested according to the Cepheid protocol. The same Xpert ultra machine was used for all the samples. Results were automatically generated in the categories negative, trace call, very low, low, medium, or high MTB (M. tuberculosis) positive by the Xpert ultra platform.

### Smear microscopy.

One milliliter of the homogenized stool was sedimented, and a smear (1 to 2 cm) was prepared and air dried. The dried smear was stained for 15 min using a 0.5% solution of auramine O (Merck, Darmstadt, Germany), decolorized for 2 min in 3% acid alcohol, and counter stained for 1 min using 0.5% potassium permanganate solution. Smears were examined within 1 h under a fluorescence microscope at ×400 magnification.

### Liquid and solid cultures.

MGIT tubes were inoculated with 500 μL of the homogenized stool samples and incubated at 37°C for a maximum of 42 days. LJ slants were inoculated with 200 μL of the resuspended stool sediments and incubated at 37°C for a maximum of 56 days. TB-positive cultures were confirmed by the presence of acid-fast bacilli upon Ziehl-Neelsen staining and the presence of antigen MPT64. The absence of acid-fast-bacillus cording and growth on blood agar was recorded as contamination. Results were reported according to International Union Against Tuberculosis and Lung Disease guidelines (53). Contaminated cultures with no definitive positive or negative result for TB were categorized as indeterminate. We note that similar MGIT and LJ culture protocols were followed.

### Statistical analyses.

Differences in baseline clinical characteristics were compared using Fisher’s exact test and Mann-Whitney U test for categorical and continuous variables, respectively. All bacterial load results (eCFU/mL) were log transformed before statistical analyses. Negative, positive, and overall (kappa scores) percentages of concordance between tests and MGIT sputum cultures were calculated using STATA version 15.1 (StataCorp, College Station, TX, USA). Sensitivity and specificity were calculated at the 95% confidence interval by using STATA version 15.1, using sputum MGIT culture as the reference test as was reported elsewhere (54). A sensitivity analysis method was used in calculation of specificity and sensitivity to minimize the interpretation bias due to contamination (54). Statistical significance was considered to be shown at a probability value of less than 0.05. Contaminated stool culture results could not be interpreted as either positive or negative. The status of these results was assessed for positivity or negativity using stool TB-MBLA, stool Xpert ultra, and stool smear, and thereafter, we categorized them as “resolved.” To ascertain whether the status of the “resolved” result was valid, we investigated the agreement with the corresponding MGIT sputum culture using kappa statistics.
